# A Combined $H_{2}$/$H_{\infty }$ Approach for Robust Joint Actuator and Sensor Fault Estimation: Application to a DC Servo-Motor System

**DOI:** 10.3390/s19112648

**Published:** 2019-06-11

**Authors:** Mariusz Buciakowski, Marcin Pazera, Marcin Witczak

**Affiliations:** Institute of Control and Computation Engineering, University of Zielona Góra, ul. Szafrana 2, 65-516 Zielona Góra, Poland

**Keywords:** fault estimation, sensor and actuator fault, robustness, estimator design, observers

## Abstract

The main objective of this paper is to develop an actuator and sensor fault estimation framework taking into account various uncertainty sources. In particular, these are divided into three groups: sensor measurement noise, process-external exogenous disturbances, as well as unknown fault dynamics. Unlike the approaches presented in the literature, here they are not processed in the same way but treated separately in a suitably tailored fashion. Finally, the approach resolves to minimizing their effect on the fault estimation error in either the $H_{2}$ or $H_{\infty }$ sense. As a result, a mixed performance–based actuator fault estimation framework is obtained, along with its convergence conditions. The final part of the paper presents performance analysis results obtained for a DC servo-motor. Subsequently, another three-tank-system-based example is presented. In both cases, the proposed approach is compared with an alternative one, which clearly exhibits its superiority.

## 1. Introduction

The problem of recovering immeasurable quantities has received considerable research attention. Indeed, it was initiated with the advent of the celebrated Kalman filter and Luenberger observer [1,2], which are traditionally applied to the state estimation of linear systems. Subsequently, the research attention was focused on unknown input observers (UIOs) [2,3,4], which were used for both control and fault diagnosis (FD) [5,6] purposes. Another important aspect pertains to the fact that UIOs can be applied in various multi-model-based configurations [7,8,9], and hence, they can model nonlinearities and tackle various multiple decoupling scenarios.

Indeed, the appealing property of UIOs pertains to the fact that they can decouple the effect of given inputs on the state estimation error. This makes it possible to achieve a state estimator insensitive to a given set of actuator faults. To tackle the problem of sensor fault detection and isolation (FDI), a bank of suitably designed observers was utilized [2]. Each of them was sensitive to all but one fault. Such a strategy allowed the formulation of suitable FDI logic capable of providing appropriate FDI decisions. Thus, FD can be perceived as a multi-level task aimed at deciding if a fault has occurred (fault detection), finding its location (fault isolation), and estimating its size (fault identification and estimation) [10]. In the literature, the FDI problem has been tackled from various angles and with a large spectrum of tools, e.g., [1,3,5,6,7,8,9,11,12,13,14,15,16,17,18]. On the other hand, fault estimation has received significantly less research attention. However, this situation is changing rapidly with the advent of an active fault-tolerant control (FTC) [3,6,19,20,21]. Indeed, active FTC requires on-line fault diagnosis including both FDI as well as fault identification and estimation. This fact becomes even more evident while dealing with the so-called integrated FTC [3,22,23], which eliminates the need for the classical FDI while relying on fault estimation exclusively. Unlike the conventional FTC, its main appealing property pertains to the fact that all fault estimation errors are incorporated into the control design framework. Similarly to observer-based output-feedback control, such a strategy requires proving the so-called separation principle [3,24]. Irrespective of the elegance of integrated FTC, it cannot fully compensate all fault estimation inaccuracies. This raises an obvious conclusion that the better the information about the fault, the better the FTC. This evident fact is the main force exciting the need for the development of more accurate and efficient fault estimation schemes. This is particularly important in the case of two crucial components of any system, namely sensors and actuators (cf. [25,26,27,28] and the references therein). However, it is an obvious fact that their estimation quality can be impaired by external exogenous process disturbances and measurement noise. To tackle this unappealing phenomenon, two main strategies can be distinguished, namely decoupling and attenuation. The former is typically based on the application of UIOs [2,3], which decouple the effect of an unknown input disturbance from the states estimation error. As a consequence, its influence on the fault estimation quality is eliminated. However, the quality of such a decoupling strongly depends on the mathematical disturbance distribution model. Another group of strategies can be used for attenuating the effect of the disturbance/noise on the estimation error. In this case, the disturbance/noise can be modeled in strictly stochastic fashion like in the case of the celebrated Kalman filter. In that event, an optimal fault estimation filter can be developed, as proposed in [29]. Its optimality is, of course, proven under a zero mean white noise assumption concerning uncertainty acting on the system. An alternative approach is to use the $H_{\infty }$ paradigm and hence impose an assumption that the uncertainty-related signal has a finite energy [30]. Using such approaches, the maximum gain between uncertainty factors and the estimation error can be minimized. Another strategy is to minimize the mean square value of such a response, which boils down to an $H_{2}$ [31] approach. Finally, the uncertain factors can be described using convex sets [32,33], and their worst-case values are taken into account while determining the estimates. However, such a strategy may lead to conservative estimates, the quality of which depends solely on precise knowledge about worst-case values of the upper and lower bounds of uncertainty factors.

The development presented in this paper is motivated by the fact that measurement noise cannot be formally threaded as a signal with a finite energy. Indeed, nowadays, such an uncertainty source can be mainly associated with the quantization error resulting from an analog-to-digital conversion. On the other hand, the process disturbance usually has an intermittent character. This observation leads to the idea of treating it in a different way. This means that the influence of measurement noise is to be minimized with the $H_{2}$ approach while the process disturbance is to be tackled with the $H_{\infty }$ one. Therefore, the proposed strategy captures benefits from both $H_{2}$ and $H_{\infty }$ paradigms. Finally, its appealing feature is that simultaneous actuator and sensor fault estimation can be performed. Thus, the contribution of the paper can be summarized as follows:

1. to propose a novel fault estimator structure capable of estimating possibly simultaneous sensor and actuator faults;
2. the proposed estimator can tackle both an exogenous process disturbance with finite energy and a random measurement noise;
3. the estimator design procedure allows the minimizing of noise/disturbance effects on both state and fault estimation errors;
4. the estimator design procedure yields a fault estimator with a guaranteed trade-off between fault and state estimation quality.

Finally, it should be noted that the proposed approach is devoted to linear systems. There are, of course, approaches that can be used to tackle fault diagnosis of nonlinear systems using a nonlinear model description directly [34,35,36]. However, the approach proposed in this paper can be extended to nonlinear systems by modeling them as linear parameter-varying (LPV) or Takagi–Sugeno ones. Subsequently, by the convexity principle [37], a direct extension of the proposed algorithm can be developed.

The paper is organized in the following order. Section 2 provides necessary preliminaries and suitable system transformation for further deliberations. Section 3 formulates the problem along with the suitable state and sensor/actuator fault estimator. It also derives the underlying estimation error along with its compact form dynamics. The section ends with definitions and supplementary lemmas. Section 4 delivers the technical theorem required for completing the design procedure (Section 6). An alternative approach is also briefly presented in Section 5. Subsequently, Section 7 exhibits an illustrative example concerning the application of the proposed strategy to a DC motor as well as a three–tank system. Finally, the last section concludes the paper.

## 2. Preliminaries

Let us consider a linear discrete-time system:

(1) $$\begin{array}{cc} x_{k+1} & =Ax_{k}+Bu_{k}+Bf_{a,k}+W_{1}w_{1,k}, \end{array}$$

(2) $$\begin{array}{cc} y_{k} & =Cx_{k}+C_{f}f_{s,k}+W_{2}w_{2,k}, \end{array}$$

where $x_{k}\in R^{n}$, $u_{k}\in R^{r}$, and $y_{k}\in R^{m}$ stand for the state, input, and output, respectively. Whilst $f_{a,k}\in R^{r}$ and $f_{s,k}\in R^{s}$ stand for the actuator and sensor faults, respectively. Vectors $w_{1,k}\in R^{q_{1}}$ and $w_{2,k}\in R^{q_{2}}$ signify system disturbance and measurement noise signals, respectively. Matrices $A\in R^{n\times n}$, $B\in R^{n\times r}$, $C\in R^{m\times n}$, $C_{f}\in R^{s}$, $W_{1}\in R^{n\times q_{1}}$, $W_{2}\in R^{m\times q_{2}}$ are constant and possess appropriate dimensions. Moreover, it is assumed that the *i*th actuator fault $f_{a,k,i}$ and *j*th sensor fault $f_{s,k,j}$ are detectable, isolable, and identifiable [19]. Finally, a fundamental assumption is that s+r≤m, which simply means that it is impossible to estimate more faults than the existing number of measurable outputs.

To make the paper self-contained, let us recall that

(3) $$\begin{array}{cc} l_{2}^{n_{w}}=\left\{w\in R_{w}^{n}{|\;∥w∥}_{l_{2}}<+\infty \right\},{∥w∥}_{l_{2}} & ={\left(\sum_{k=0}^{\infty }{∥w_{k}∥}^{2}\right)}^{\frac{1}{2}}. \end{array}$$

Bearing in mind the above nomenclature, let us impose the following assumptions:

- *Assumption 1:* The process of exogenous disturbance is bounded in the $l_{2}^{q_{1}}$ sense, i.e., $w_{1,k}\in l_{2}^{q_{1}}$;
- *Assumption 2:* The measurement noise $w_{2,k}$ is a random sequence;
- *Assumption 3:* Actuator and sensor faults’ rates of change $\varepsilon_{a,k}=f_{a,k+1}-f_{a,k}$, $\varepsilon_{s,k}=f_{s,k+1}-f_{s,k}$ are bounded in the $l_{2}^{r}$ and $l_{2}^{s}$ sense, i.e., $\varepsilon_{a,k}\in l_{2}^{r}$ and $\varepsilon_{s,k}\in l_{2}^{s}$, respectively.

*Assumption 1* signifies that the exogenous disturbance $w_{1,k}$ has a finite energy. Contrarily, $w_{2,k}$ does not satisfy this property. Indeed, inaccuracies of today’s digital sensors can be accompanied by quantization errors and other random factors that can be efficiently exemplified as a random sequence. This clearly necessitates *Assumption 2*. Furthermore, any physical actuator exhibits a finite performance, which cannot be arbitrarily increased. Thus, it is natural to assume that the fault rate of change cannot increase without limits as well. Indeed, while a given fault settles, it converges to zero, and hence, in most of the works presented in the literature [38], $\varepsilon_{a,k}=0$ (or $\dot{f}=0$ in the continuous-time framework). Using a similar reasoning, a sensor fault rate of change cannot increase without limits as well.

Given the general description of the system along with assumptions, the system (1) and (2) is to be converted into a form that is more suitable for further deliberations. Indeed, it couples the state and sensor fault into an extended-state vector and hence eliminates its direct existence in the output equation. Unlike the sensor fault, the actuator fault is estimated by applying an adaptive rule.

Let ${\bar{x}}_{k}={\left[x_{k}^{T},\;f_{s,k}^{T}\right]}^{T}$, then the system (1) and (2) can be transformed into the so-called descriptor shaped by:

(4) $$\begin{array}{cc} \left[\begin{array}{cc} I & 0\\ 0 & 0 \end{array}\right]\left[\begin{array}{c} x_{k+1}\\ f_{s,k+1} \end{array}\right] & =\left[\begin{array}{cc} A & 0\\ 0 & I \end{array}\right]\left[\begin{array}{c} x_{k}\\ f_{s,k} \end{array}\right]+\left[\begin{array}{c} B\\ 0 \end{array}\right]u_{k}+\left[\begin{array}{c} B\\ 0 \end{array}\right]f_{a,k}+\left[\begin{array}{c} 0\\ I \end{array}\right]\varepsilon_{s,k}+\left[\begin{array}{c} W1\\ 0 \end{array}\right]w_{1,k}, \end{array}$$

(5) $$\begin{array}{cc} y_{k} & =\left[\begin{array}{cc} C & C_{f} \end{array}\right]\left[\begin{array}{c} x_{k}\\ f_{s,k} \end{array}\right]+W_{2}w_{2,k}, \end{array}$$

which can be rewritten in a simpler form:

(6) $$\begin{array}{cc} \bar{E}{\bar{x}}_{k+1} & =\bar{A}{\bar{x}}_{k}+\bar{B}u_{k}+\bar{B}f_{a,k}+\bar{H}\varepsilon_{s,k}+{\bar{W}}_{1}w_{1,k}, \end{array}$$

(7) $$\begin{array}{cc} y_{k} & =\bar{C}{\bar{x}}_{k}+W_{2}w_{2,k}. \end{array}$$

Given the general description of the system, the underlying simultaneous actuator and sensor fault estimation problem is to be tackled in the subsequent section.

## 3. Problem Formulation

This section describes the main problems that will be investigated in this paper. Let us consider a discrete-time estimator of the form

(8) $$\begin{array}{cc} z_{k+1} & =Nz_{k}+Mu_{k}+Ly_{k}+T_{1}\bar{B}{\hat{f}}_{a,k}, \end{array}$$

(9) $$\begin{array}{cc} {\hat{\bar{x}}}_{k} & =z_{k}+T_{2}y_{k}, \end{array}$$

(10) $$\begin{array}{cc} {\hat{f}}_{a,k+1} & ={\hat{f}}_{a,k}+F\left(y_{k}-\bar{C}{\hat{\bar{x}}}_{k}\right), \end{array}$$

where ${\hat{\bar{x}}}_{k}\in R^{n}$ and ${\hat{f}}_{k}\in R^{r}$ denote the estimate of the state and actuator fault, respectively. Thus, the estimator design problem amounts to determining the gain matrices N, M, L, and F, while the form of $T_{1}$ and $T_{2}$ is to be exposed in a direct form. Based on (6) and (7), the state estimation error ${\bar{e}}_{k}$ obeys

(11) $$\begin{array}{cc} {\bar{e}}_{k}={\bar{x}}_{k}-{\hat{\bar{x}}}_{k} & ={\bar{x}}_{k}-z_{k}-T_{2}\bar{C}{\bar{x}}_{k}-T_{2}W_{2}w_{2,k}=\left(I-T_{2}\bar{C}\right){\bar{x}}_{k}-z_{k}-T_{2}W_{2}w_{2,k}\\ & =T_{1}\bar{E}{\bar{x}}_{k}-z_{k}-T_{2}W_{2}w_{2,k}. \end{array}$$

From (11) it can be deduced that

(12) $$z_{k}=T_{1}\bar{E}{\bar{x}}_{k}-{\bar{e}}_{k}-T_{2}W_{2}w_{2,k}.$$

Thus, bearing in mind (4)–(5) and (11)–(12), the dynamics of the estimation error obeys

(13) $$\begin{array}{cc} {\bar{e}}_{k+1} & =T_{1}\bar{E}{\bar{x}}_{k+1}-z_{k+1}-T_{2}W_{2}w_{2,k+1}=T_{1}\bar{A}{\bar{x}}_{k}+T_{1}\bar{B}u_{k}+T_{1}\bar{B}f_{a,k}\\ & +T_{1}\bar{H}\varepsilon_{s,k}+T_{1}{\bar{W}}_{1}w_{1,k}-Nz_{k}-Mu_{k}-L\bar{C}{\bar{x}}_{k}-LW_{2}w_{2,k}\\ & -T_{1}\bar{B}{\hat{f}}_{a,k}-T_{2}W_{2}w_{2,k+1}=\left(T_{1}\bar{A}-NT_{1}\bar{E}-L\bar{C}\right){\bar{x}}_{k}+T_{1}\bar{B}e_{a,k}\\ & +\left(T_{1}\bar{B}-M\right)u_{k}+N{\bar{e}}_{k}+T_{1}\bar{H}\varepsilon_{s,k}+T_{1}{\bar{W}}_{1}w_{1,k}\\ & -T_{2}W_{2}w_{2,k+1}+\left(NT_{2}-L\right)W_{2}w_{2,k}. \end{array}$$

Based on (13), it is apparent that the following relationships must hold:

(14) $$\begin{array}{cc} T_{1}\bar{B}-M & =0, \end{array}$$

(15) $$\begin{array}{cc} T_{1}\bar{A}-NT_{1}\bar{E}-L\bar{C} & =0. \end{array}$$

Thus, it is evident that:

(16) $$\begin{array}{cc} M & =T_{1}\bar{B}, \end{array}$$

(17) $$\begin{array}{cc} T_{1}\bar{A} & =NT_{1}\bar{E}+L\bar{C}, \end{array}$$

and hence

(18) $$\begin{array}{cc} T_{1}\bar{A} & =N\left(I-T_{2}\bar{C}\right)+L\bar{C}, \end{array}$$

(19) $$\begin{array}{cc} T_{2}\bar{A} & =N-NT_{2}\bar{C}+L\bar{C}, \end{array}$$

(20) $$\begin{array}{cc} N & =T_{1}\bar{A}+NT_{2}\bar{C}-L\bar{C}, \end{array}$$

(21) $$\begin{array}{cc} N & =T_{1}\bar{A}-\left(L-NT_{2}\right)\bar{C}, \end{array}$$

(22) $$\begin{array}{cc} N & =T_{1}\bar{A}-K\bar{C}, \end{array}$$

where the new gain matrices are:

(23) $$\begin{array}{cc} K & =L-NT_{2}, \end{array}$$

(24) $$\begin{array}{cc} L & =K+NT_{2}. \end{array}$$

As a consequence, the augmented estimation error is given as

(25) $$\begin{array}{c} {\bar{e}}_{k+1}=\left(T_{1}\bar{A}-K\bar{C}\right){\bar{e}}_{k}+T_{1}\bar{B}e_{a,k}+T_{1}{\bar{W}}_{1}w_{1,k}+T_{1}\bar{H}\varepsilon_{s,k}-T_{2}W_{2}w_{2,k+1}-KW_{2}w_{k}. \end{array}$$

Subsequently, the dynamics of the estimation error obeys

(26) $$\begin{array}{c} \begin{array}{cc} e_{a,k+1}=f_{a,k+1}+{\hat{f}}_{a,k+1} & =f_{a,k+1}+f_{a,k}-f_{a,k}-{\hat{f}}_{a,k}-F\left(y_{k}-\bar{C}{\hat{x}}_{k}\right)\\ & =\varepsilon_{a,k}+e_{a,k}-F\bar{C}{\bar{e}}_{k}-FW_{2}w_{2,k}, \end{array} \end{array}$$

with $\varepsilon_{a,k}=f_{a,k+1}-f_{a,k}$. For the purpose of further deliberations, let us define the following vectors

(27) $$\begin{array}{c} {\tilde{e}}_{k}={\left[{\bar{e}}_{k}^{T},\;e_{a,k}^{T}\right]}^{T},\;{\tilde{\varepsilon }}_{k}={\left[\varepsilon_{s,k}^{T},\;\varepsilon_{a,k}^{T}\right]}^{T}, \end{array}$$

which make it possible to describe (25) and (26) as follows

(28) $$\begin{array}{cc} {\tilde{e}}_{k+1} & =\left(\tilde{A}-\tilde{K}\tilde{C}\right){\bar{e}}_{k}+{\tilde{W}}_{1}w_{1,k}-\tilde{K}{\tilde{W}}_{2}w_{2,k}+\tilde{H}w_{2,k+1}+\tilde{E}{\tilde{\varepsilon }}_{k}, \end{array}$$

(29) $$\begin{array}{cc} e_{f,k} & =\tilde{I}{\tilde{e}}_{k}, \end{array}$$

where

$$\begin{array}{cc} \tilde{A} & =\left[\begin{array}{cc} T_{2}\bar{A} & T_{1}\bar{B}\\ 0 & I \end{array}\right],\;\tilde{K}=\left[\begin{array}{c} K\\ F \end{array}\right],\;\tilde{C}=\left[\begin{array}{cc} \bar{C} & 0 \end{array}\right],\;{\tilde{W}}_{1}=\left[\begin{array}{c} T_{1}{\bar{W}}_{1}\\ 0 \end{array}\right],\;{\tilde{W}}_{2}=W_{2},\\ \tilde{H} & =\left[\begin{array}{c} -T_{2}W_{2}\\ 0 \end{array}\right],\;\tilde{E}=\left[\begin{array}{cc} T_{1}\bar{H} & 0\\ 0 & I \end{array}\right],\;\tilde{I}={\left[\begin{array}{cc} 0 & I \end{array}\right]}^{T}. \end{array}$$

Based on the above transformations, five different objectives are considered while determining the gain matrices K and L for (8)–(10): (i) asymptotic convergence to zero of the state and fault estimation error (28) and (29); (ii) rejection of $w_{1,k}$; (iii) rejection of $\varepsilon_{k}$ in the $H_{\infty }$ sense; (iv) rejection of $w_{2,k}$ in the $H_{2}$ sense; and (v) rejection of $w_{2,k+1}$ in the $H_{2}$ sense. The above problem is formulated formally through the following definition:

**Definition** **1.** *The system *(8)–(10)* is robustly convergent in the $H_{2}/H_{\infty }$ sense if given scalars $\mu_{1}>0$, $\mu_{2}>0$, $\gamma_{1}>0$ and $\gamma_{2}>0$:*

*(i)*  (8)–(10) *are asymptotically stable when $w_{1,k}=0$, $w_{2,k}=0$, $w_{2,k+1}=0$, and ${\tilde{\varepsilon }}_{k}=0$;*
*(ii)*  *$∥G_{e_{f}w_{1}}{\left(z\right)∥}_{\infty }<\mu_{1}$ when $w_{1,k}\neq 0$;*
*(iii)*  *$∥G_{e_{f}\tilde{\varepsilon }}{\left(z\right)∥}_{\infty }<\mu_{2}$ when ${\tilde{\varepsilon }}_{k}\neq 0$;*
*(iv)*  *$∥G_{e_{f}w_{2}}{\left(z\right)∥}_{2}<\gamma_{1}$ when $w_{2,k}\neq 0$;*
*(v)*  $∥G_{e_{f}w_{2}^{*}}{\left(z\right)∥}_{2}<\gamma_{2}$ when $w_{2,k+1}\neq 0$,

*where*

$$\begin{array}{cc} G_{e_{f}w_{1}}\left(z\right) & =\tilde{I}{\left(zI-\left(\tilde{A}-\tilde{K}\tilde{C}\right)\right)}^{-1}{\tilde{W}}_{1},\\ G_{e_{f}\tilde{\varepsilon }}\left(z\right) & =\tilde{I}{\left(zI-\left(\tilde{A}-\tilde{K}\tilde{C}\right)\right)}^{-1}\tilde{E},\\ G_{e_{f}w_{2}}\left(z\right) & =\tilde{I}{\left(zI-\left(\tilde{A}-\tilde{K}\tilde{C}\right)\right)}^{-1}\left(-\tilde{K}{\tilde{W}}_{2}\right),\\ G_{e_{f}w_{2}^{*}}\left(z\right) & =\tilde{I}{\left(zI-\left(\tilde{A}-\tilde{K}\tilde{C}\right)\right)}^{-1}\tilde{H}, \end{array}$$

*denotes the transfer function from the inputs $w_{1,k}$, ${\tilde{\varepsilon }}_{k}$, $w_{2,k}$, and $w_{2,k+1}$ to the output $e_{f,k}$, respectively.*

For further deliberations, let us consider the following system

(30) $$\begin{array}{cc} X_{k+1} & =AX_{k}+BU_{k}, \end{array}$$

(31) $$\begin{array}{cc} Y_{k} & =CX_{k}, \end{array}$$

with the transfer function matrix $G\left(z\right)=C{\left(zI-A\right)}^{-1}B$. Subsequently, let us remind the following lemmas:

**Lemma** **1.** *${∥G\left(z\right)∥}_{2}<\gamma^{2}$ if, and only if, there exist matrices P≻0 and W≻0 such that $Trace\left(W\right)<\gamma^{2}$ and*

(32) $$\begin{array}{c} \left[\begin{array}{ccc} -P & * & *\\ A^{T}P & -P & *\\ B^{T}P & 0 & -I \end{array}\right]≺0,\;\left[\begin{array}{cc} W & *\\ C^{T} & P \end{array}\right]≻0. \end{array}$$

**Lemma** **2.** *${∥G\left(z\right)∥}_{\infty }<\mu$ if, and only if, there exist matrices P≻0 such that*

(33) $$\begin{array}{c} \left[\begin{array}{cccc} -P & * & * & *\\ A^{T}P & -P & * & *\\ B^{T}P & 0 & -\mu I & *\\ 0 & C & 0 & -\mu I \end{array}\right]≺0. \end{array}$$

**Proof.**  For proof of *Lemma 1* and *Lemma 2*, the reader is referred to [39]. □

**Lemma** **3.** *${∥G\left(z\right)∥}_{\infty }<\mu^{2}$ if, and only if, there exist matrices P≻0 such that*

(34) $$\begin{array}{c} \left[\begin{array}{cccc} -P & * & * & *\\ 0 & -I & * & *\\ A^{T}P & C^{T} & -P & *\\ B^{T}P & 0 & 0 & -\mu_{2}^{2}I \end{array}\right]≺0. \end{array}$$

**Proof.**  Proof of *Lemma 3* can be obtained using the authors’ results concerning fault estimation [40]. □

Given Definition 1 and the general structure of the estimator, along with its estimation error (28) and (29), the design procedure for determining the gain matrices K and F will be given in the next section.

## 4. Fault Estimator Design

The objective of this section is to determine necessary and sufficient conditions for the synthesis of the observer (8)–(10) for the system (1) and (2), satisfying the conditions expressed by Definition 1. Using the above-mentioned definitions, it is possible to formulate the main result of this section:

**Theorem** **1.** *For prescribed attenuation levels $\mu_{1}>0$, $\mu_{2}>0$, $\gamma_{1}>0$, and $\gamma_{2}>0$ of $w_{1,k}$, ${\tilde{\varepsilon }}_{k}$, $w_{2,k}$, and $w_{2,k+1}$, respectively, the $H_{2}/H_{\infty }$ estimator design problem is solvable if, and only if, there exist P≻0, $V_{1}≻0$, $V_{2}≻0$ and $\tilde{N}$ such that the following conditions are satisfied:*

(35) $$\begin{array}{c} \left[\begin{array}{ccc} -P & * & *\\ {\tilde{A}}^{T}P-{\tilde{C}}^{T}{\tilde{N}}^{T} & -P & *\\ {\tilde{W}}_{2}^{T}{\tilde{N}}^{T} & 0 & -I \end{array}\right]≺0, \end{array}$$

(36) $$\begin{array}{c} \left[\begin{array}{ccc} -P & * & *\\ {\tilde{A}}^{T}P-{\tilde{C}}^{T}{\tilde{N}}^{T} & -P & *\\ -{\tilde{H}}^{T}P & 0 & -I \end{array}\right]≺0, \end{array}$$

(37) $$\begin{array}{c} \left[\begin{array}{cc} V_{1} & *\\ {\tilde{I}}^{T} & P \end{array}\right]≻0, \end{array}$$

(38) $$\begin{array}{c} \left[\begin{array}{cc} V_{2} & *\\ {\tilde{I}}^{T} & P \end{array}\right]≻0, \end{array}$$

(39) $$\begin{array}{c} Trace\left(V_{1}\right)<\gamma_{1}^{2} \end{array}$$

(40) $$\begin{array}{c} Trace\left(V_{2}\right)<\gamma_{2}^{2} \end{array}$$

(41) $$\begin{array}{c} \left[\begin{array}{cccc} -P & * & * & *\\ 0 & -I & * & *\\ {\tilde{A}}^{T}P-{\bar{C}}^{T}{\tilde{N}}^{T} & {\tilde{I}}^{T} & -P & *\\ {\tilde{W}}_{1}^{T}P & 0 & 0 & -\mu_{1}^{2}I \end{array}\right]≺0, \end{array}$$

(42) $$\begin{array}{c} \left[\begin{array}{cccc} -P & * & * & *\\ 0 & -I & * & *\\ {\tilde{A}}^{T}P-{\tilde{C}}^{T}{\tilde{N}}^{T} & {\tilde{I}}^{T} & -P & *\\ {\tilde{E}}^{T}P & 0 & 0 & -\mu_{2}^{2}I \end{array}\right]≺0, \end{array}$$

*where $\tilde{N}=P\tilde{K}$.*

**Proof.**  The above LMIs were obtained by performing a set of suitable manipulations on the matrices underlying (28) and (29), which can be summarized as follows:

**Constraint** (35)**:** Setting $(\tilde{A}-\tilde{K}\tilde{C})\to A$, ${\tilde{W}}_{2}\to B$, P→P in *Lemma 1* gives
  (43) $$\begin{array}{c} \left[\begin{array}{ccc} -P & * & *\\ {\tilde{A}}^{T}P-{\tilde{C}}^{T}{\tilde{K}}^{T}P & -P & *\\ {\tilde{W}}_{2}^{T}{\tilde{K}}^{T}P & 0 & -I \end{array}\right]≺0. \end{array}$$
  Substituting $\tilde{N}=P\tilde{K}$ gives (35).
**Constraint** (36)**:** Setting $(\tilde{A}-\tilde{K}\tilde{C})\to A$, $\tilde{H}\to B$, P→P in *Lemma 1*,
  (44) $$\begin{array}{c} \left[\begin{array}{ccc} -P & * & *\\ {\tilde{A}}^{T}P-{\tilde{C}}^{T}{\tilde{K}}^{T}P & -P & *\\ -{\tilde{H}}^{T}P & 0 & -I \end{array}\right]≺0. \end{array}$$
  Substituting $\tilde{N}=P\tilde{K}$ gives (36).
**Constraint** (37)–(40)**:** Setting P→P, $V_{1}\to W$, $V_{2}\to W$, $\bar{I}\to C$ in *Lemma 1* gives (37) and (38), respectively.
**Constraint** (41)**:** Setting $(\tilde{A}-\tilde{K}\tilde{C})\to A$, ${\tilde{W}}_{1}\to B$, P→P, $\bar{I}\to C$ in *Lemma 3* gives
  (45) $$\begin{array}{c} \left[\begin{array}{cccc} -P & * & * & *\\ 0 & -I & * & *\\ {\tilde{A}}^{T}P-{\bar{C}}^{T}{\tilde{K}}^{T}P & {\tilde{I}}^{T} & -P & *\\ {\tilde{W}}_{1}^{T}P & 0 & 0 & -\mu_{1}^{2}I \end{array}\right]≺0. \end{array}$$
  Using $\tilde{N}=P\tilde{K}$ gives (41).
**Constraint** (42)**:** Setting $(\tilde{A}-\tilde{K}\tilde{C})\to A$, $\tilde{E}\to B$, P→P, $\bar{I}\to C$ in *Lemma 3*,
  (46) $$\begin{array}{c} \left[\begin{array}{cccc} -P & * & * & *\\ 0 & -I & * & *\\ {\tilde{A}}^{T}P-{\tilde{C}}^{T}{\tilde{K}}^{T}P & {\tilde{I}}^{T} & -P & *\\ {\tilde{E}}^{T}P & 0 & 0 & -\mu_{2}^{2}I \end{array}\right]≺0. \end{array}$$
  Using $\tilde{N}=P\tilde{K}$ gives (42).

□

## 5. An Alternative Approach to Fault Estimator Design

Before proceeding to the performance validation results, let us introduce an alternative approach, which is based on a direct application of the approach proposed in [39]. An alternative approach is characterized by the following theorem:

**Theorem** **2.** *For prescribed attenuation levels $\mu_{1}>0$, $\mu_{2}>0$, $\gamma_{1}>0$, and $\gamma_{2}>0$ of $w_{1,k}$, ${\tilde{\varepsilon }}_{k}$, $w_{2,k}$, and $w_{2,k+1}$, respectively, the $H_{2}/H_{\infty }$ estimator design problem is solvable if, and only if, there exist P≻0, $V_{1}≻0$, $V_{2}≻0$ and $\tilde{N}$ such that the following conditions are satisfied:*

(47) $$\begin{array}{c} \left[\begin{array}{ccc} -P & * & *\\ {\tilde{A}}^{T}P-{\tilde{C}}^{T}{\tilde{N}}^{T} & -P & *\\ {\tilde{W}}_{2}^{T}{\tilde{N}}^{T} & 0 & -I \end{array}\right]≺0, \end{array}$$

(48) $$\begin{array}{c} \left[\begin{array}{ccc} -P & * & *\\ {\tilde{A}}^{T}P-{\tilde{C}}^{T}{\tilde{N}}^{T} & -P & *\\ -{\tilde{H}}^{T}P & 0 & -I \end{array}\right]≺0, \end{array}$$

(49) $$\begin{array}{c} \left[\begin{array}{cc} V_{1} & *\\ {\tilde{I}}^{T} & P \end{array}\right]≻0, \end{array}$$

(50) $$\begin{array}{c} \left[\begin{array}{cc} V_{2} & *\\ {\tilde{I}}^{T} & P \end{array}\right]≻0, \end{array}$$

(51) $$\begin{array}{c} Trace\left(V_{1}\right)<\gamma_{1}^{2} \end{array}$$

(52) $$\begin{array}{c} Trace\left(V_{2}\right)<\gamma_{2}^{2} \end{array}$$

(53) $$\begin{array}{c} \left[\begin{array}{cccc} -P & * & * & *\\ {\tilde{A}}^{T}P-{\bar{C}}^{T}{\tilde{N}}^{T} & -P & * & *\\ {\tilde{W}}_{1}^{T}P & 0 & -\mu_{1}I & *\\ 0 & \tilde{I} & 0 & -\mu_{1}I \end{array}\right]≺0, \end{array}$$

(54) $$\begin{array}{c} \left[\begin{array}{cccc} -P & * & * & *\\ {\tilde{A}}^{T}P-{\bar{C}}^{T}{\tilde{N}}^{T} & -P & * & *\\ E^{T}P & 0 & -\mu_{2}I & *\\ 0 & \tilde{I} & 0 & -\mu_{2}I \end{array}\right]≺0, \end{array}$$

*where $\tilde{N}=P\tilde{K}$.*

**Proof.**  The proof can be performed based on [39], which boils down to:

**Constraint** (47)**:** Setting $(\tilde{A}-\tilde{K}\tilde{C})\to A$, ${\tilde{W}}_{2}\to B$, P→P in *Lemma 1* gives
  (55) $$\begin{array}{c} \left[\begin{array}{ccc} -P & * & *\\ {\tilde{A}}^{T}P-{\tilde{C}}^{T}{\tilde{K}}^{T}P & -P & *\\ {\tilde{W}}_{2}^{T}{\tilde{K}}^{T}P & 0 & -I \end{array}\right]≺0. \end{array}$$
  Using $\tilde{N}=P\tilde{K}$ gives (47).
**Constraint** (36)**:** Setting $(\tilde{A}-\tilde{K}\tilde{C})\to A$, $\tilde{H}\to B$, P→P in *Lemma 1*,
  (56) $$\begin{array}{c} \left[\begin{array}{ccc} -P & * & *\\ {\tilde{A}}^{T}P-{\tilde{C}}^{T}{\tilde{K}}^{T}P & -P & *\\ -{\tilde{H}}^{T}P & 0 & -I \end{array}\right]≺0. \end{array}$$
  Using $\tilde{N}=P\tilde{K}$ gives (48).
**Constraint** (49)–(52)**:** Setting P→P, $V_{1}\to W$, $V_{2}\to W$, $\bar{I}\to C$ in *Lemma 1* gives (49)–(50), respectively.
**Constraint** (53)**:** Setting $(\tilde{A}-\tilde{K}\tilde{C})\to A$, ${\tilde{W}}_{1}\to B$, P→P, $\bar{I}\to C$ in *Lemma 2* gives
  (57) $$\begin{array}{c} \left[\begin{array}{cccc} -P & * & * & *\\ {\tilde{A}}^{T}P-{\bar{C}}^{T}{\tilde{K}}^{T}P & -P & * & *\\ {\tilde{W}}_{1}^{T}P & 0 & -\mu_{1}I & *\\ 0 & \tilde{I} & 0 & -\mu_{1}I \end{array}\right]≺0. \end{array}$$
  Using $\tilde{N}=P\tilde{K}$ gives (53).
**Constraint** (42)**:** Setting $(\tilde{A}-\tilde{K}\tilde{C})\to A$, $\tilde{E}\to B$, P→P, $\bar{I}\to C$ in *Lemma 2*,
  (58) $$\begin{array}{c} \left[\begin{array}{cccc} -P & * & * & *\\ {\tilde{A}}^{T}P-{\bar{C}}^{T}{\tilde{K}}^{T}P & -P & * & *\\ E^{T}P & 0 & -\mu_{2}I & *\\ 0 & \tilde{I} & 0 & -\mu_{2}I \end{array}\right]≺0. \end{array}$$
  Using $\tilde{N}=P\tilde{K}$ gives (54).

□

## 6. Final Design Procedure of the Fault Estimation Scheme

The problem of determining the estimator gain matrices described by Theorem 1 can be treated as an optimization problem directed at minimizing the disturbance attenuation levels $\mu_{1}$, $\mu_{2}$, $\gamma_{1}$, and $\gamma_{2}$. Setting $\beta_{1}=\gamma_{1}^{2}$ and $\beta_{2}=\gamma_{2}^{2}$, the structure of the whole observer design can be summarized by the following algorithm:

**Offline computation:**
  1. Iteratively change the values of $\mu_{1}$ and $\mu_{2}$.
  2. Solve the optimization problem
    (59) $$\begin{array}{c} \mathrm{minimize}\;\beta_{1}+\beta_{2} \end{array}$$
    (60) $$\begin{array}{c} \left\{\begin{array}{cc} \mathrm{subject}\;\mathrm{to}\;(35)–(42) & \mathrm{for}\;\mathrm{the}\;\mathrm{proposed}\;\mathrm{approach}\\ \mathrm{subject}\;\mathrm{to}\;(47)–(54) & \;\mathrm{for}\;\mathrm{the}\;\mathrm{alternative}\;\mathrm{approach} \end{array}\right. \end{array}$$
    to find a trade-off between disturbance attenuation levels $\gamma_{1}$, $\gamma_{2}$, $\mu_{1}$, and $\mu_{2}$, where $\gamma_{1}=\sqrt{\beta_{1}}$ and $\gamma_{2}=\sqrt{\beta_{2}}$.
  3. If the attenuation levels are not satisfactory, then go to Step 1, or else obtain matrices K and F and calculate:
    (61) $$\begin{array}{cc} \left[\begin{array}{cc} T_{1} & T_{2} \end{array}\right] & ={\left[\begin{array}{c} \bar{E}\\ \bar{C} \end{array}\right]}^{†}, \end{array}$$
    (62) $$\begin{array}{cc} N & =T_{1}\bar{A}-K\bar{C}, \end{array}$$
    (63) $$\begin{array}{cc} M & =T_{1}\bar{B} \end{array}$$
    (64) $$\begin{array}{cc} L & =K+NT_{2}. \end{array}$$
**Online computation:**
  1. Compute the fault estimates ${\hat{f}}_{a,k}$ and ${\hat{f}}_{s,k}$ with (8)–(10).

## 7. Illustrative Examples

This section presents an empirical verification of the proposed approach. For that purpose, a DC servo-motor given in [40] and a three-tank system where considered. For the DC motor, the system matrices were as follows:

$$\begin{array}{cc} A & =\left[\begin{array}{ccc} 1.0000 & 0.1000 & 0\\ 0 & 0.8495 & 0.4977\\ 0 & -0.0357 & 0.9995 \end{array}\right],\;B=\left[\begin{array}{c} 0\\ 0\\ 0.0729 \end{array}\right],\\ C_{f} & =\left[\begin{array}{c} 0\\ 1 \end{array}\right],\;C=\left[\begin{array}{ccc} 1 & 0 & 0\\ 0 & 1 & 0 \end{array}\right],\;W_{1}=\left[\begin{array}{c} 0.05\\ 0\\ 0 \end{array}\right],\;W_{2}=\left[\begin{array}{cc} 0.025 & 0\\ 0 & 0.025 \end{array}\right], \end{array}$$

while for the three–tank system they are given by:

$$\begin{array}{cc} A & =\left[\begin{array}{ccc} 0.9982 & 0 & 0\\ 0.0018 & 0.9973 & 0\\ 0 & 0.0025 & 0.9972 \end{array}\right],\;B=\left[\begin{array}{c} 11.14\\ 0\\ 0 \end{array}\right],\\ C_{f} & =\left[\begin{array}{c} 0\\ 1 \end{array}\right],\;C=\left[\begin{array}{ccc} 1 & 0 & 0\\ 0 & 0 & 1 \end{array}\right],\;W_{1}=\left[\begin{array}{c} 0.05\\ 0\\ 0 \end{array}\right],\;W_{2}=\left[\begin{array}{cc} 0.025 & 0\\ 0 & 0.025 \end{array}\right]. \end{array}$$

### 7.1. Analysis of $H_{2}/H_{\infty }$ Trade-off—DC Servo-Motor

**Figure 1:**
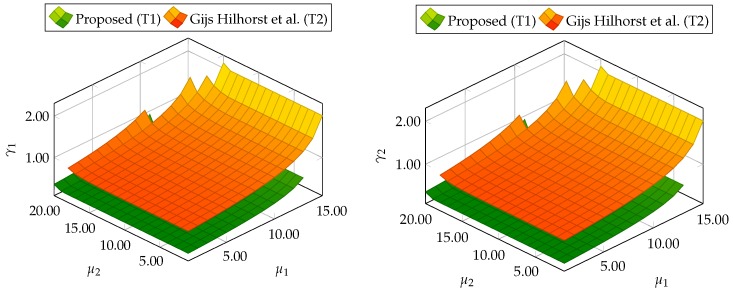
Trade-off between $∥G_{e_{f}w_{1}}{\left(z\right)∥}_{\infty }<\mu_{1}$, $∥G_{e_{f}\tilde{\varepsilon }}{\left(z\right)∥}_{\infty }<\mu_{2}$, $∥G_{e_{f}w_{2}}{\left(z\right)∥}_{2}<\gamma_{1}$, and $∥G_{e_{f}w_{2}}{\left(z\right)∥}_{2}<\gamma_{2}$ for proposed Theorem 1 (T1) and Theorem 2 (T2) based on [39].

At the beginning, the trade-off between $∥G_{e_{f}w_{1}}{\left(z\right)∥}_{\infty }<\mu_{1}$, $∥G_{e_{f}\tilde{\varepsilon }}{\left(z\right)∥}_{\infty }<\mu_{2}$, $∥G_{e_{f}w_{2}}{\left(z\right)∥}_{2}<\gamma_{1}$, and $∥G_{e_{f}w_{2}}{\left(z\right)∥}_{2}<\gamma_{2}$ depending on $\mu_{1}$ and $\mu_{2}$ is shown. In particular, 20 values of $\mu_{1}$ and $\mu_{2}$ were selected from a range between 1 to 20. Next, for each value of $\mu_{1}$ and $\mu_{2}$, the optimization problem (59) was solved under constraints (35)–(42) for the proposed approach and constraints (47)–(54) for the alternative approach. Figure 1 shows the evolution of $\gamma_{1}$ and $\gamma_{2}$ for different $\mu_{1}$ and $\mu_{2}$. From these results, it can be deduced that the proposed approach (Theorem 1) allows us to obtain lower values of $\gamma_{1}$ and $\gamma_{2}$ than results obtained using Theorem 2. Moreover, Table 1 contains the minimal values of $\mu_{1}$ and $\mu_{2}$ for which the constraints (59) are satisfied.

### 7.2. Simulation Case—DC Servo-Motor

In the simulation case, the input signal is chosen as a step signal with an amplitude of 10 [V]. The initial conditions for the system and the observer are:

$$\begin{array}{cc} x_{0} & ={\left[0,\;0,\;0\right]}^{T},\;z_{0}={\left[0.2,\;0.1,\;0.5\right]}^{T},\;{\hat{f}}_{a,0}=0,\;{\hat{f}}_{s,0}=0. \end{array}$$

**Figure 2:**
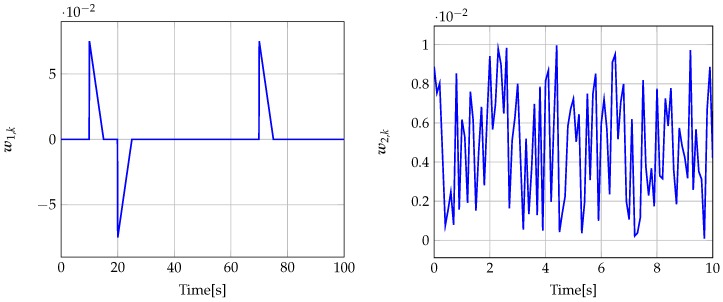
Disturbance signal $w_{1,k}$ (**left**) (for t=0,…,100) and measurement noise signal $w_{2,k}$ (**right**) (for t=0,…,10).

The signal $w_{1,k}$ is shown in Figure 2. $w_{2,k}$ was chosen as a uniformly distributed random vector, where each element takes values in the interval [−0.01,0.01]. The estimator parameters calculated for $\mu_{1}$ and $\mu_{2}$ given in Table 1 are

$$\begin{array}{cc} N & =\left[\begin{array}{cccc} -0.7279 & 0.0750 & 0 & 0.0250\\ -21.8327 & 1.8572 & 0.5000 & 1.0082\\ -17.6055 & 1.0311 & 0.9996 & 1.0668\\ 21.8327 & -1.8572 & -0.5000 & -1.0083 \end{array}\right],\;M=\left[\begin{array}{c} 0\\ 0\\ 0.0729\\ 0 \end{array}\right],\;L=\left[\begin{array}{cc} 0.8639 & -0.0000\\ 10.9163 & 0\\ 8.8028 & 0\\ -10.9164 & 0 \end{array}\right],\\ T_{1} & =\left[\begin{array}{cccc} 0.5 & 0 & 0 & 0\\ 0 & 1 & 0 & 0\\ 0 & 0 & 1 & 0\\ 0 & -1 & 0 & 0 \end{array}\right],\;T_{2}=\left[\begin{array}{cc} 0.5 & 0\\ 0 & 0\\ 0 & 0\\ 0 & 1 \end{array}\right]\;F=\left[\begin{array}{cc} 35.2003 & -3.6162 \end{array}\right],\\ N & =\left[\begin{array}{cccc} -0.5637 & 0.0658 & 0 & 0.0158\\ -16.0557 & 1.6293 & 0.5000 & 0.7804\\ -8.7495 & 0.5343 & 0.9996 & 0.5701\\ 16.0558 & -1.6293 & -0.5000 & -0.7803 \end{array}\right],\;M=\left[\begin{array}{c} 0\\ 0\\ 0.0729\\ 0 \end{array}\right],\;L=\left[\begin{array}{cc} 0.7818 & 0\\ 8.0279 & 0\\ 4.3748 & 0\\ -8.0279 & 0 \end{array}\right],\\ T_{1} & =\left[\begin{array}{cccc} 0.5 & 0 & 0 & 0\\ 0 & 1 & 0 & 0\\ 0 & 0 & 1 & 0\\ 0 & -1 & 0 & 0 \end{array}\right],\;T_{2}=\left[\begin{array}{cc} 0.5 & 0\\ 0 & 0\\ 0 & 0\\ 0 & 1 \end{array}\right]\;F=\left[\begin{array}{cc} 14.3768 & -1.6188 \end{array}\right], \end{array}$$

for Theorem 1 and Theorem 2, respectively. For the purpose of further comparative study, two DC servo-motor fault scenarios (FS) were considered:

**FS1**  $$\begin{array}{cc} f_{a,k}= & \left\{\begin{array}{cc} -0.2 & 50\leq t\leq 55,\\ 0 & \mathrm{otherwise}. \end{array}\right.\\ f_{s,k}= & 0 \end{array}$$
**FS2**  $$\begin{array}{cc} f_{a,k}= & 0\\ f_{s,k}= & \left\{\begin{array}{cc} -0.3 & 50\leq t\leq 55,\\ 0 & \mathrm{otherwise}. \end{array}\right. \end{array}$$
**FS3**  $$\begin{array}{cc} f_{a,k}= & \left\{\begin{array}{cc} -0.2 & 50\leq t\leq 55,\\ 0 & \mathrm{otherwise}. \end{array}\right.\\ f_{s,k}= & \left\{\begin{array}{cc} -0.3 & 50\leq t\leq 55,\\ 0 & \mathrm{otherwise}. \end{array}\right. \end{array}$$

**Figure 3:**
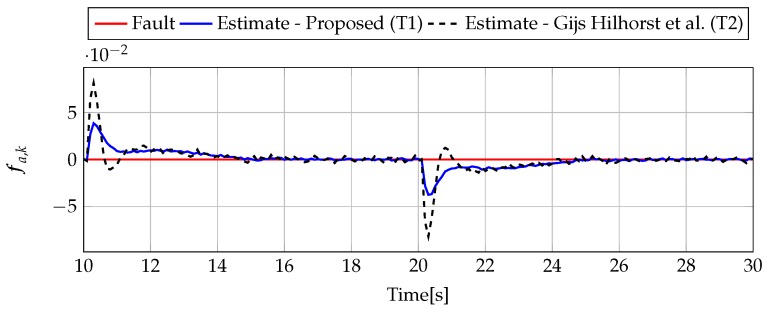
Actuator fault estimate for the fault–free case (for t=10,…,30) with Theorem 1 (T1) and Theorem 2 (T2), based on [39] for disturbance signal $w_{1,k}$.

**Figure 4:**
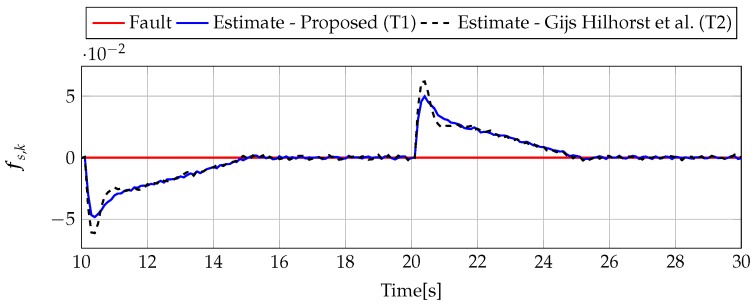
Sensor fault estimate for the fault-free case (for t=10,…,30) with Theorem 1 (T1) and Theorem 2 (T2) based on [39] for disturbance signal $w_{1,k}$.

**Figure 5:**
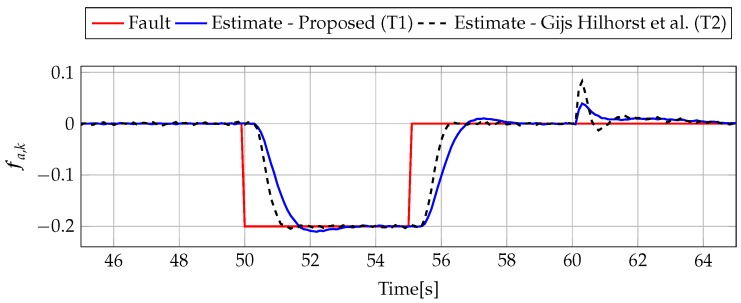
Fault $f_{a,k}$ (red line) and fault estimate obtained with Theorem 1 (T1) and Theorem 2 (T2) based on [39] for FS1 (for t=45,…,65[s]) as well as the response for disturbance signal $w_{1,k}$.

**Figure 6:**
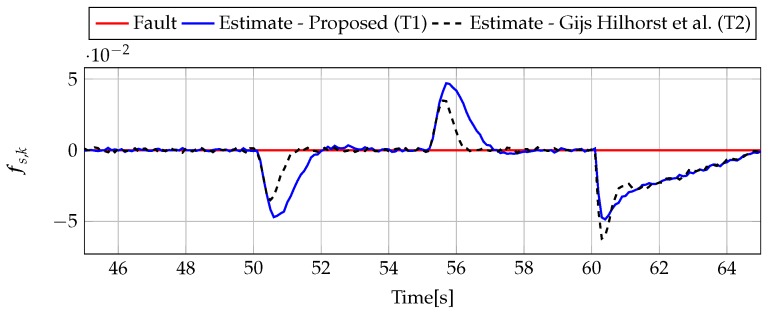
Fault $f_{s,k}$ (red line) and fault estimate obtained with Theorem 1 (T1) and Theorem 2 (T2) based on [39] for FS1 (for t=45,…,65 [s]) as well as the response for disturbance signal $w_{1,k}$.

**Figure 7:**
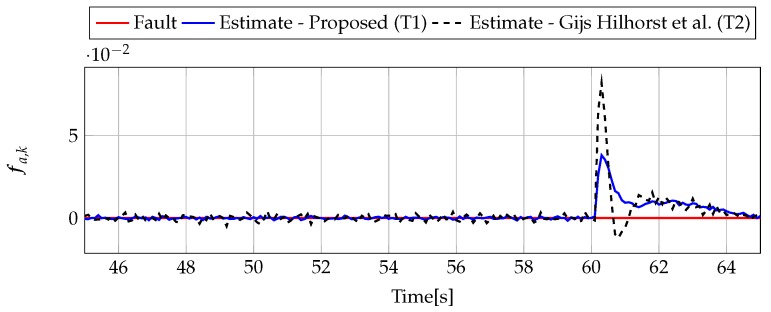
Fault $f_{a,k}$ (red line) and fault estimate obtained with Theorem 1 (T1) and Theorem 2 (T2) based on [39] for FS2 (for t=45,…,65 [s]) as well as the response for disturbance signal $w_{1,k}$.

**Figure 8:**
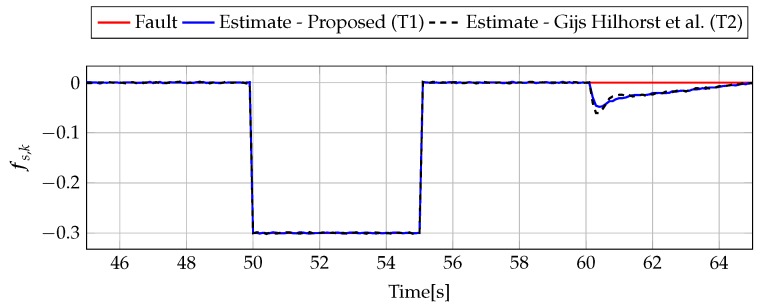
Fault $f_{s,k}$ (red line) and fault estimate obtained with Theorem 1 (T1) and Theorem 2 (T2) based on [39] for FS2 (for t=45,…,65 [s]) as well as the response for disturbance signal $w_{1,k}$.

**Figure 9:**
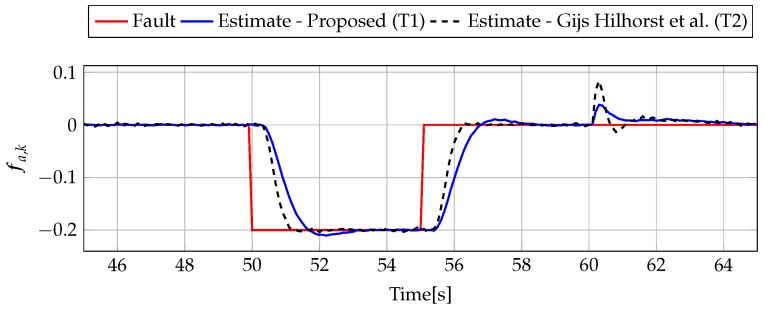
Fault $f_{a,k}$ (red line) and fault estimate obtained with Theorem 1 (T1) and Theorem 2 (T2) based on [39] for FS3 (for t=45,…,65 [s]) as well as the response for disturbance signal $w_{1,k}$.

**Figure 10:**
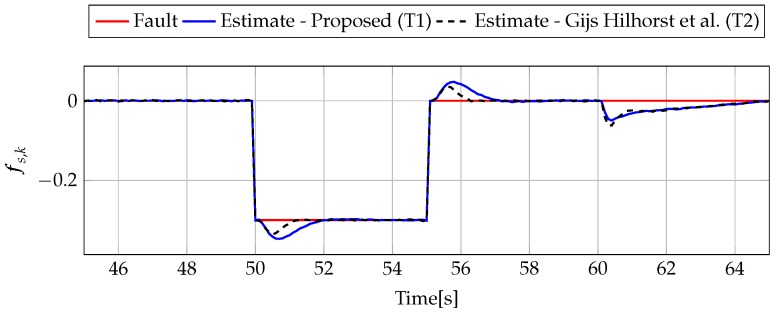
Fault $f_{s,k}$ (red line) and fault estimate obtained with Theorem 1 (T1) and Theorem 2 (T2) based on [39] for FS3 (for t=45,…,65 [s]) as well as the response for disturbance signal $w_{1,k}$.

The analysis of fault estimation starts with a fault-free case ($f_{s,k}=0$, $f_{s,k}=0$). Figure 3 and Figure 4 show the response of the fault estimate to the disturbance signal $w_{1,k}$. From these results, it can be seen that the response of the fault estimate (Theorem 1) has a smaller amplitude than for the parameters obtained using Theorem 2. In particular, in the case of actuator fault estimation, better performance results from the fact that the $H_{\infty }$ norm ($\mu_{1}$) of the transfer function $G_{e_{f}w_{1}}\left(z\right)$ is 0.9595, which means that disturbances are rejected correctly, in contrast to the results obtained using Theorem 2, where the $H_{\infty }$ norm of transfer function $G_{e_{f}w_{1}}\left(z\right)$ is 2.2283. Subsequently, Figure 5, Figure 6, Figure 7, Figure 8, Figure 9 and Figure 10 present the real values of faults (solid red line) and their estimation obtained using Theorem 1 (solid blue line) and Theorem 2 (dashed black line) for FS1–FS3. Additionally, the response to the disturbance signal $w_{1,k}$ at time t=60,…,65 [s] was included as well. It can be observed that the response to $w_{1,k}$ is similar to a fault-free case. Furthermore, fault estimation is performed with a good quality as well. However, it can be seen that the convergence of the fault estimation is not as good as for parameters calculated using Theorem 2. To summarize, the estimator obtained with Theorem 1 is able to reject the disturbances better than the one using Theorem 2. This is, of course, realized at the expense of the fault estimation convergence. Finally, Figure 5, Figure 6, Figure 9 and Figure 10 illustrate that if actuator and sensor faults act in the same time-range, then they can be estimated simultaneously. However, it is natural that at the transient phase they influence each other.

### 7.3. Analysis of $H_{2}/H_{\infty }$ Trade-off—Three-Tank System

**Figure 11:**
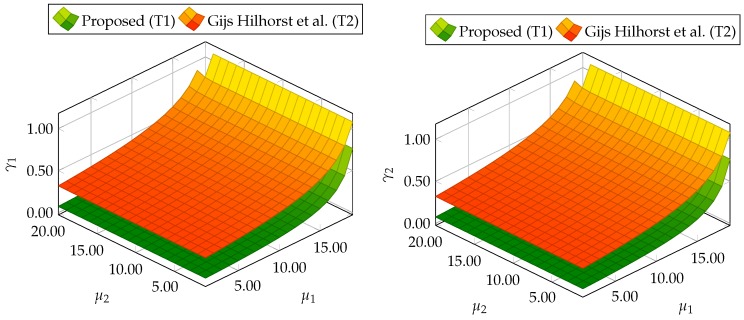
Trade-off between $∥G_{e_{f}w_{1}}{\left(z\right)∥}_{\infty }<\mu_{1}$, $∥G_{e_{f}\tilde{\varepsilon }}{\left(z\right)∥}_{\infty }<\mu_{2}$, $∥G_{e_{f}w_{2}}{\left(z\right)∥}_{2}<\gamma_{1}$, and $∥G_{e_{f}w_{2}}{\left(z\right)∥}_{2}<\gamma_{2}$ for proposed Theorem 1 (T1) and Theorem 2 (T2) based on [39].

Similarly for the DC servo-motor example, 20 values of $\mu_{1}$ and $\mu_{2}$ were selected from a range between 1 and 20 to present the trade-off between $∥G_{e_{f}w_{1}}{\left(z\right)∥}_{\infty }<\mu_{1}$, $∥G_{e_{f}\tilde{\varepsilon }}{\left(z\right)∥}_{\infty }<\mu_{2}$, $∥G_{e_{f}w_{2}}{\left(z\right)∥}_{2}<\gamma_{1}$, and $∥G_{e_{f}w_{2}}{\left(z\right)∥}_{2}<\gamma_{2}$ for the three-tank system. For each value of $\mu_{1}$ and $\mu_{2}$, the optimization problem (59) was solved under constraints. Figure 11 shows the evolution of $\gamma_{1}$ and $\gamma_{2}$ for different $\mu_{1}$ and $\mu_{2}$. Finally, Table 2 contains the minimal values of $\mu_{1}$ and $\mu_{2}$ for which the constraints (59) are satisfied. Thus, it is evident that this example also proves the superiority of the proposed approach with respect to the comparative one.

### 7.4. Simulation Case—Three-Tank System

In the simulation case, the input signal is chosen as a step signal with an amplitude of 0.0001 [m${}^{3}$/s]. The linear model along with all its parameters are derived from [41]. The initial conditions for the system and the observer are:

$$\begin{array}{cc} x_{0} & ={\left[0,\;0,\;0\right]}^{T},\;z_{0}={\left[0.02,\;0.01,\;0.05\right]}^{T},\;{\hat{f}}_{a,0}=0,\;{\hat{f}}_{s,0}=0. \end{array}$$

**Figure 12:**
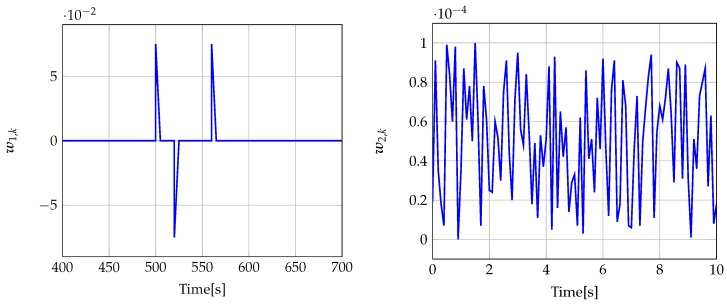
Disturbance signal $w_{1,k}$ (**left**) (for t=400,…,700) and measurement noise signal $w_{2,k}$ (**right**) (for t=0,…,10).

The signal $w_{1,k}$ is shown in Figure 12. $w_{2,k}$ was chosen as a uniformly distributed random vector, where each element takes values in the interval [−0.0001,0.0001]. The estimator parameters calculated for $\mu_{1}$ and $\mu_{2}$ are included in Table 2.

$$\begin{array}{cc} N & =\left[\begin{array}{cccc} -1.4333 & 0 & 0.0001 & 0.0001\\ 0.0000 & 0.9973 & 0.0000 & 0.0000\\ 0.0000 & 0.0025 & 0.9972 & 0.0000\\ 0.0000 & -0.0025 & 0.9971 & 0.0001 \end{array}\right],\;M=\left[\begin{array}{c} 5.5700\\ 0\\ 0\\ 0 \end{array}\right],\;L=\left[\begin{array}{cc} 1.2157 & 0.0000\\ 0.0018 & 0.0000\\ 0.0000 & 0.0000\\ 0.0000 & 0.0000 \end{array}\right],\\ T_{1} & =\left[\begin{array}{cccc} 0.5000 & 0 & 0 & 0\\ 0 & 1.0000 & 0 & 0\\ 0 & 0 & 1.0000 & 0\\ 0 & 0 & -1.0000 & 0 \end{array}\right],\;T_{2}=\left[\begin{array}{ccc} 0.5000 & 0\\ 0 & 0\\ 0 & 0.00000 & 1.0000 \end{array}\right]\;F=\left[\begin{array}{cc} 0.2230 & 0.0000 \end{array}\right],\\ N & =\left[\begin{array}{cccc} -1.3283 & 0 & 0.0000 & 0.0000\\ 0.0000 & 0.9973 & 0.0000 & 0.0000\\ 0.0000 & 0.0025 & 0.9972 & 0.0000\\ 0.0000 & -0.0025 & -0.9974 & -0.0002 \end{array}\right],\;M=\left[\begin{array}{c} 5.5700\\ 0\\ 0\\ 0 \end{array}\right],\;L=\left[\begin{array}{cc} 1.1633 & 0\\ 0.0018 & -0.0000\\ -0.0000 & -0.0000\\ 0.0000 & 0.0000 \end{array}\right],\\ T_{1} & =\left[\begin{array}{cccc} 0.5000 & 0 & 0 & 0\\ 0 & 1.0000 & 0 & 0\\ 0 & 0 & 1.0000 & 0\\ 0 & 0 & -1.0000 & 0 \end{array}\right],\;T_{2}=\left[\begin{array}{cc} 0.5000 & 0\\ 0 & 0\\ 0 & -0.0000\\ 0 & 1.0000 \end{array}\right]\;F=\left[\begin{array}{cc} 0.2385 & 0.0000 \end{array}\right], \end{array}$$

for Theorem 1 and Theorem 2, respectively. For the purpose of further comparative study, the following three-tank system fault scenarios (FS) were considered:

**FS1**  $$\begin{array}{cc} f_{a,k}= & \left\{\begin{array}{cc} -0.0002 & 550\leq t\leq 555,\\ 0 & \mathrm{otherwise}. \end{array}\right.\\ f_{s,k}= & 0 \end{array}$$
**FS2**  $$\begin{array}{cc} f_{a,k}= & 0\\ f_{s,k}= & \left\{\begin{array}{cc} -0.0003 & 550\leq t\leq 555,\\ 0 & \mathrm{otherwise}. \end{array}\right. \end{array}$$
**FS3**  $$\begin{array}{cc} f_{a,k}= & \left\{\begin{array}{cc} -0.0002 & 550\leq t\leq 555,\\ 0 & \mathrm{otherwise}. \end{array}\right.\\ f_{s,k}= & \left\{\begin{array}{cc} -0.0003 & 550\leq t\leq 555,\\ 0 & \mathrm{otherwise}. \end{array}\right. \end{array}$$

**Figure 13:**
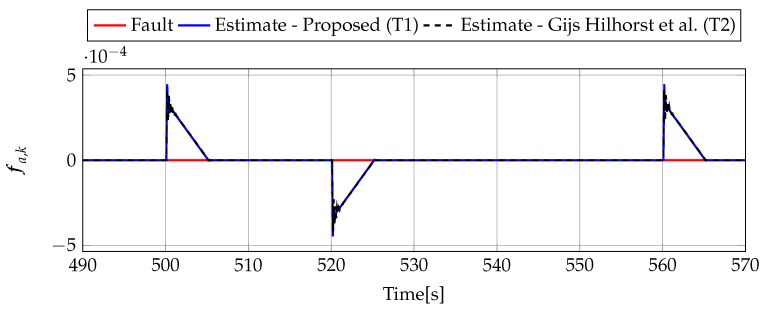
Actuator fault estimate for fault-free case (for t=490,…,570) with Theorem 1 (T1) and Theorem 2 (T2) based on [39] for disturbance signal $w_{1,k}$.

**Figure 14:**
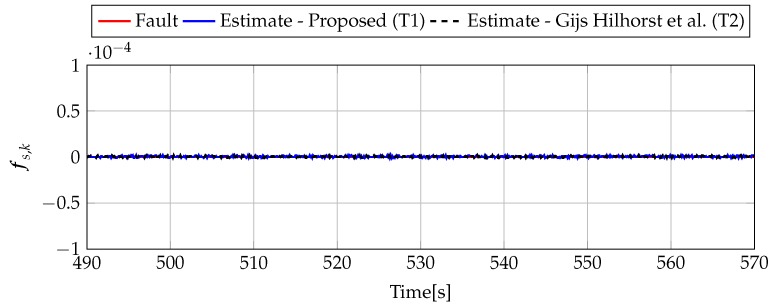
Sensor fault estimate for fault-free case (for t=490,…,570) with Theorem 1 (T1) and Theorem 2 (T2) based on [39] for disturbance signal $w_{1,k}$.

**Figure 15:**
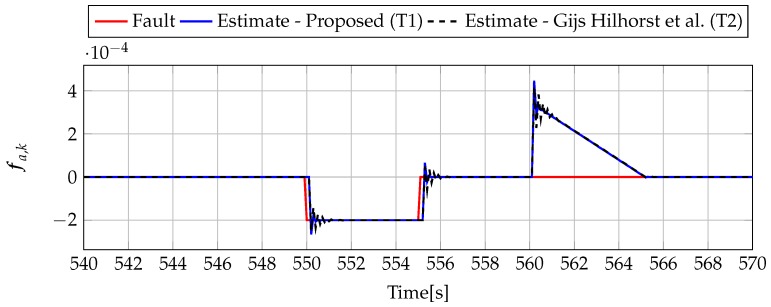
Fault $f_{a,k}$ (red line) and fault estimate obtained with Theorem 1 (T1) and Theorem 2 (T2) based on [39] for FS1 (for t=490,…,570 [s]) as well as the response for disturbance signal $w_{1,k}$.

**Figure 16:**
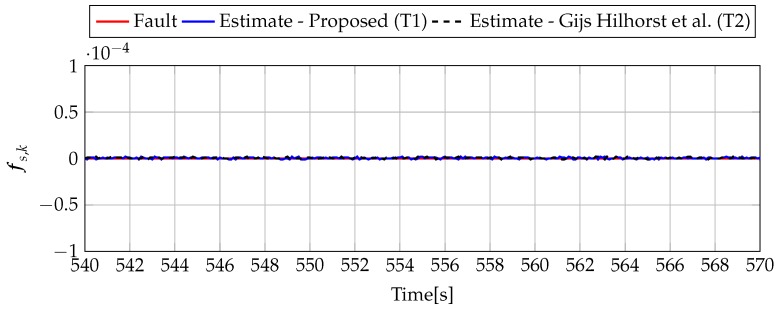
Fault $f_{s,k}$ (red line) and fault estimate obtained with Theorem 1 (T1) and Theorem 2 (T2) based on [39] for FS1 (for t=540,…,570 [s]) as well as the response for disturbance signal $w_{1,k}$.

**Figure 17:**
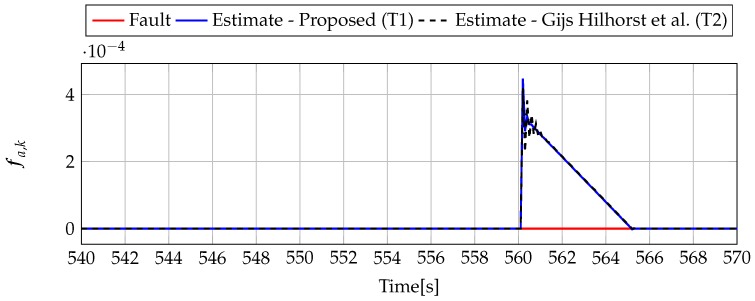
Fault $f_{a,k}$ (red line) and fault estimate obtained with Theorem 1 (T1) and Theorem 2 (T2) based on [39] for FS2 (for t=540,…,570 [s]) as well as the response for disturbance signal $w_{1,k}$.

**Figure 18:**
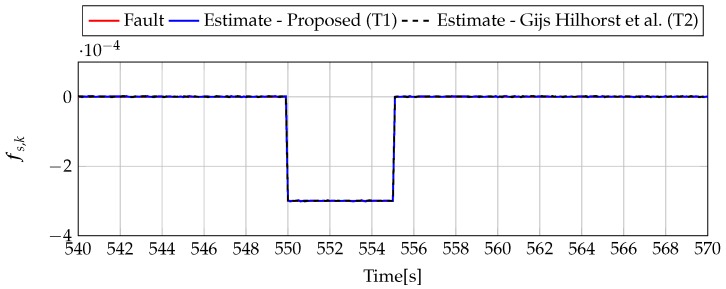
Fault $f_{s,k}$ (red line) and fault estimate obtained with Theorem 1 (T1) and Theorem 2 (T2) based on [39] for FS2 (for t=540,…,570 [s]) as well as the response for disturbance signal $w_{1,k}$.

**Figure 19:**
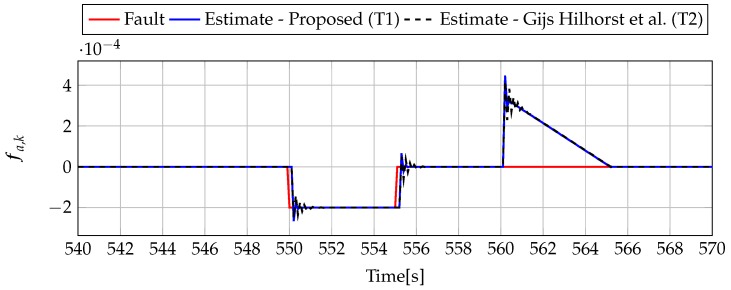
Fault $f_{a,k}$ (red line) and fault estimate obtained with Theorem 1 (T1) and Theorem 2 (T2) based on [39] for FS3 (for t=540,…,570 [s]) as well as the response for disturbance signal $w_{1,k}$.

**Figure 20:**
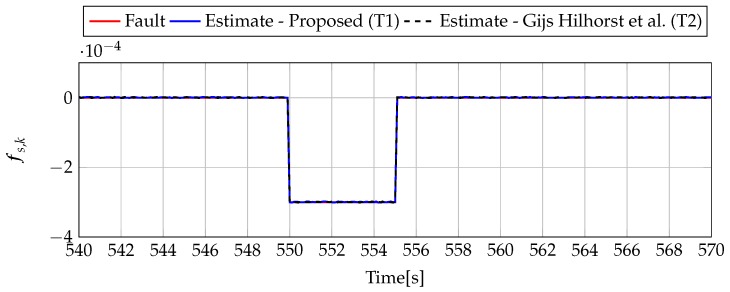
Fault $f_{s,k}$ (red line) and fault estimate obtained with Theorem 1 (T1) and Theorem 2 (T2) based on [39] for FS3 (for t=540,…,570 [s]) as well as the response for disturbance signal $w_{1,k}$.

Analogously to the previous example, let us start an analysis with a fault-free case ($f_{s,k}=0$, $f_{s,k}=0$). Figure 13 and Figure 14 show the response of the fault estimate to the disturbance signal $w_{1,k}$. It can be seen that the difference in rejecting $w_{1,k}$ between the observer designed using Theorem 1 and Theorem 2 is not as visible as in the first example. However, the response for an observer designed using Theorem 1 has a smaller oscillation than for the one designed using Theorem 2. Next, Figure 15, Figure 16, Figure 17, Figure 18, Figure 19 and Figure 20 present the real values of the fault (solid red line) and its estimation obtained using Theorem 1 (solid blue line) and Theorem 2 (dashed black line) for FS1–FS3. Additionally, the response to the disturbance signal $w_{1,k}$ at time t=560,…,565 [s] was included as well. It can be observed that the response to $w_{1,k}$ is similar to a fault-free case. Furthermore, fault estimation is performed with a good quality as well.

## 8. Conclusions

The main research problem addressed in this paper was oriented toward developing an actuator and sensor fault estimation capable of delivering fault estimates under various sources of uncertainty, namely sensor measurement noise, process-external exogenous disturbances, as well as unknown fault dynamics. Unlike the approaches presented in the literature, these were not treated in the same way but analyzed separately in a tailored fashion. As a result, a complete design procedure was obtained along with a suitable convergence analysis. The final part of the paper presents the performance of the proposed approach using DC-motor and three-tank system benchmarks, as well as a comparative study with an alternative strategy. The presented comparison clearly justifies the superiority of the proposed scheme. Future research directions are oriented toward applying the proposed scheme within an integrated FTC framework. Another appealing research avenue pertains to extending the proposed strategy toward a class of nonlinear systems.

## Figures and Tables

**Table 1:** The minimal values of $\mu_{1}$ and $\mu_{2}$ for which the constraints (59) are satisfied.

	Proposed (T1)	Hilhorst et al. (T2)
$\gamma_{1}$	0.3193	1.6467
$∥G_{e_{f}w_{2}}{\left(z\right)∥}_{2}$	0.5666	1.1870
$\gamma_{1}-{∥G_{e_{f}w_{2}}\left(z\right)∥}_{2}$	0.3510	0.4597
$\gamma_{2}$	0.9176	0.6547
$∥G_{e_{f}w_{2}}{\left(z\right)∥}_{2}$	0.3227	1.6467
$\gamma_{2}-{∥G_{e_{f}w_{2}}\left(z\right)∥}_{2}$	0.5948	0.9920
$\mu_{1}$	1.0000	3.2999
$∥G_{e_{f}w_{1}}{\left(z\right)∥}_{\infty }$	0.9595	2.2283
$\mu_{1}-{∥G_{e_{f}w_{1}}\left(z\right)∥}_{\infty }$	0.0404	1.0716
$\mu_{2}$	10.0000	10.0000
$∥G_{e_{f}\tilde{\varepsilon }}{\left(z\right)∥}_{\infty }$	9.6806	7.41762
$\mu_{2}-{∥G_{e_{f}\tilde{\varepsilon }}\left(z\right)∥}_{\infty }$	0.3193	2.5823

**Table 2:** The minimal values of $\mu_{1}$ and $\mu_{2}$ for which the constraints (59) are satisfied.

	Proposed (T1)	Hilhorst et al. (T2)
$\gamma_{1}$	0.7658	0.7701
$∥G_{e_{f}w_{2}}{\left(z\right)∥}_{2}$	0.0079	0.0089
$\gamma_{1}-{∥G_{e_{f}w_{2}}\left(z\right)∥}_{2}$	0.7579	0.7611
$\gamma_{2}$	0.7658	0.7701
$∥G_{e_{f}w_{2}}{\left(z\right)∥}_{2}$	0.0251	0.0252
$\gamma_{2}-{∥G_{e_{f}w_{2}}\left(z\right)∥}_{2}$	0.7406	0.7449
$\mu_{1}$	0.0700	0.7000
$∥G_{e_{f}w_{1}}{\left(z\right)∥}_{\infty }$	0.0088	0.0148
$\mu_{1}-{∥G_{e_{f}w_{1}}\left(z\right)∥}_{\infty }$	0.0611	0.6851
$\mu_{2}$	2.0000	4.0000
$∥G_{e_{f}\tilde{\varepsilon }}{\left(z\right)∥}_{\infty }$	1.7528	1.9587
$\mu_{2}-{∥G_{e_{f}\tilde{\varepsilon }}\left(z\right)∥}_{\infty }$	0.2471	2.0412
